# Individual, collective and contextual dimensions of sustainable lifestyle change in daily life contexts: an integrated perspective

**DOI:** 10.3389/fpsyg.2025.1505676

**Published:** 2025-01-30

**Authors:** Eugenio De Gregorio, Giuseppe Carrus, Christian Andreas Klöckner, Erica Löfström, Lassi Similä, Michael Brenner-Fliesser

**Affiliations:** ^1^Department of Health Sciences, Link Campus University, Rome, Italy; ^2^Department of Education, Roma Tre University, Rome, Italy; ^3^Department of Psychology, Norwegian University of Science and Technology, Trondheim, Norway; ^4^VTT Technical Research Centre of Finland, Espoo, Finland; ^5^LIFE Research Group, Joanneum Research Forschungsgesellschaft mbH, Graz, Austria

**Keywords:** sustainability, lifestyles, individual factors, collective factors, contextual factors

## Abstract

This paper aims to develop a conceptual and theoretical perspective on sustainable lifestyle change according to a multidisciplinary approach. In particular, we discuss the interplay between three orders of factors that, according to the literature and to our conceptual model, are relevant in shaping sustainable lifestyles and lifestyle change in people daily life contexts, such as their living neighbourhoods. The three orders of factors are the following: (1) Individual level factors (as typically present and discussed in the environmental psychology literature; e.g., attitudes, values, beliefs, intentions, emotions, connection to nature, etc.); (2) Collective level factors (as typically present and discussed in the social psychology and sociology literature; e.g., social capital, social norms, social and place identity, sense of community, place attachment, energy memories & energy cultures); (3) Contextual factors (as typically present and discussed in the environmental science and economic literature; e.g., regulations, technology, infrastructures, economic resources, etc.). For each of these three levels, we will present and discuss some classical and recent literature findings, and we will provide a summary of the current state of the art knowledge about sustainable lifestyle adoption in neighbourhoods’ contexts.

## 1 Introduction

This paper aims to present an integrated view from different perspectives on the construct of “lifestyles” with particular reference to everyday life contexts and small communities. We have examined a number of theories and models from different disciplinary fields in the human sciences (psychology, sociology, anthropology, economy and law) in order to identify what the definitions of the construct of “lifestyles”; within a small community or might be.

As for the concept of “lifestyle,” following the recent work by Schwarzinger et al. (2024), we can assume that it has an interesting history, as it can be tracked back to the early works of the German philosopher and sociologist Simmel (2004, 2023). The term “lifestyle,” which is used to distinguish it from simple behaviour to mean a broader range of human activities. Is therefore intended to highlight interconnected patterns of action in specific human life contexts, such as cities or neighbourhoods. In the context of sustainability studies, this ‘lifestyle’ perspective could include, for example, a range of individual behaviours such as energy use for appliances, space heating or mobility, dietary behaviours or other health-preserving actions. Research on “lifestyles” typically pursues the goal of understanding behaviours and their determinants in detail. The more contemporary literature also recognises that lifestyles are strongly shaped by structural conditions (e.g., mobility infrastructure, availability of certain products) that align many specific behaviours (Böhme et al., 2022). The adjective ‘sustainable’ in this context denotes a set of habits and patterns of behaviour embedded in a society and facilitated by institutions, norms and infrastructures that frame individual choices to ensure that natural resource use and waste generation are within the regenerative and assimilative capacities of ecosystems (Schwarzinger et al., 2024).

Such work on defining “lifestyles” is preliminary to application in different contexts where lifestyles are a consequence or cause of specific behaviours. Consequently, we propose a model that incorporates different levels of analysis (stemming from different research perspectives) to better understand the main triggers for sustainable lifestyle change.

We argue that a crucial point highlighted across many studies is how lifestyle is not a stable and structural condition but develops as a process that changes over time, taking into account the complex interdependence between different orders of factors at the basis of human choices and actions.

According to these premises, the concept of sustainable lifestyle was addressed in the CLEANcultures international project, funded by multiple national agencies in the context of the JPI Climate “Solstice” programme. The aim of the project is to increase understanding about local climate-related challenges, by triggering and to empowering local actors toward bottom-up activities. Additionally, the project aims at informing policymakers about local perspectives that should be considered to increase acceptance of climate policies. Within the project, the focus of the analysis is on neighbourhoods. Case studies were conducted in four countries and nine different neighbourhoods that range from suburbs to small rural communities. The project shares a methodological framework which has been adapted to local conditions and needs (see Klöckner et al., 2024 for more details on the findings). The project was conducted according to a multidisciplinary approach, reflected in an international research team sharing a professional and research interest in the study of pro-environmental behaviour, with backgrounds ranging across social, behavioral technological and engineering sciences.

In this paper, we will attempt to describe the interaction and integration between these factors, which at different levels of complexity contribute to shape the lifestyle of an individual.

**Figure 1:**
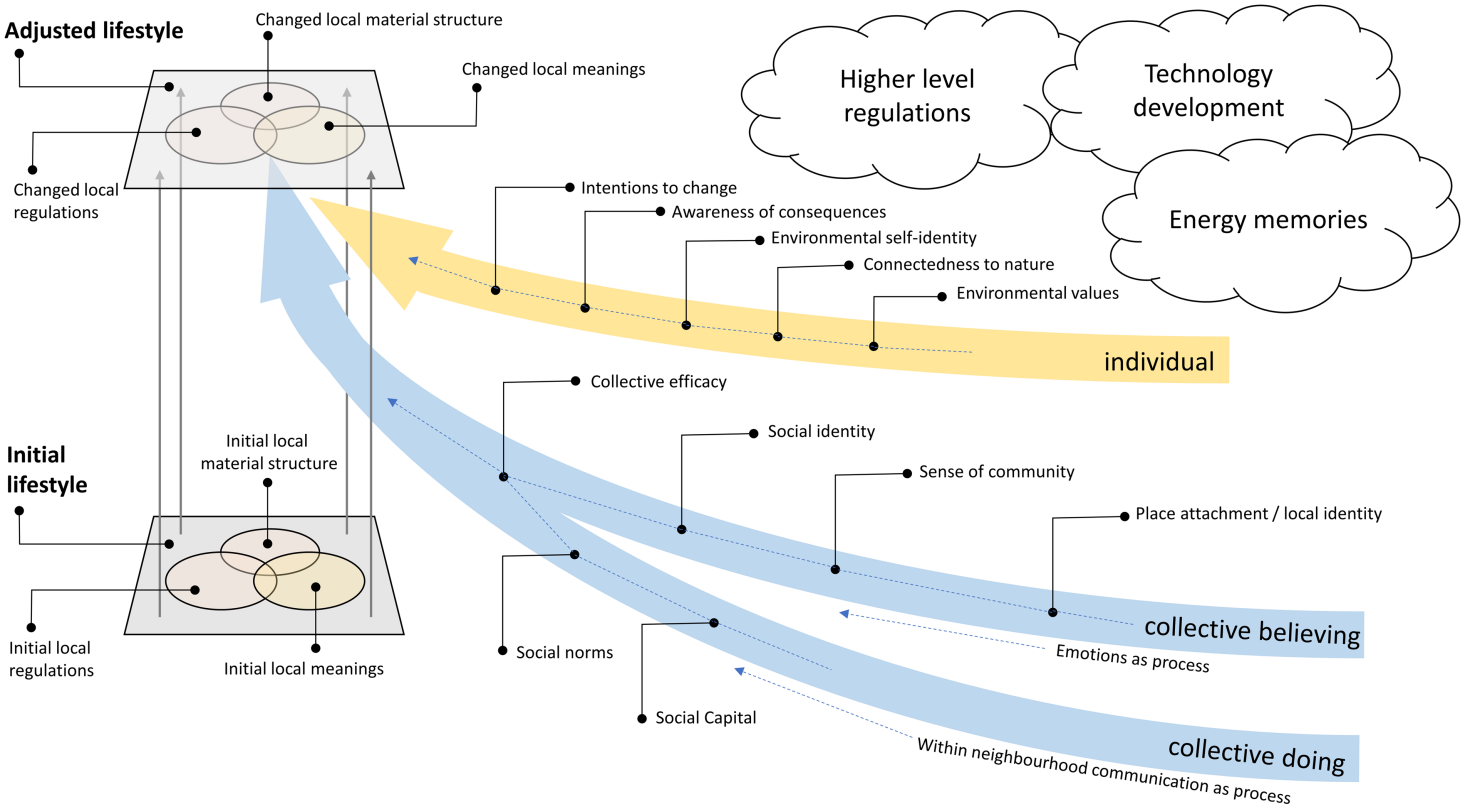
A framework of initiating lifestyle change on the neighbourhood level.

The three theoretical dimensions that contribute to an integrated perspective on the construct of “lifestyles” are described below (see Figure 1).

## 2 The proposed model

### 2.1 Individual factors

A number of scholars frequently considered individual factors such as environmental attitudes, pro-environmental values, awareness of the consequences of one’s behaviour and intentions to adopt sustainable behaviour as potential antecedents of sustainable practises and behaviour.

We will first give a brief overview of these concepts and then offer a perspective that tries to integrate individual and social aspects and understand lifestyles in neighbourhoods in their complexity.

Attitudes represent key variables in the exploration of a broad spectrum of human behaviour, including pro-environmental behaviour. Attitude is “a relatively enduring organisation of beliefs, feelings and behavioural tendencies toward socially significant objects, groups, events or symbols” (Hogg and Vaughan, 2022, p. 156) and can be explained as a positive or negative evaluation of a behaviour, based on the reasoned weight of the expected costs and benefits of that particular course of action. More specifically to the environmental domain, Fielding and Hornsey (2016) defined attitudes toward sustainable behaviour as evaluations or mental dispositions influenced by an individual’s social identity and group norms. These attitudes determine the degree of engagement in pro-environmental behaviour and are shaped by the perception of belonging to groups that share pro-environmental values. Social identity theory (referred to in the next section) highlights how a sense of collective responsibility and group dynamics can promote sustainable behaviour. With regard to sustainable lifestyles, attitudes here can be defined as the set of psychological and behavioural dispositions that favour the adoption of sustainable practises, influenced by personal, cultural and social factors. Matharu et al. (2021) emphasise the role of health- and sustainability-oriented lifestyle choices in promoting responsible consumption and participation in shared economy models, stimulated by social norms and collective goals for sustainability. Similarly, Bassi (2023) considers attitudes toward sustainable lifestyles to be the set of consumers’ perceptions, beliefs and predispositions toward practises that promote environmental sustainability. The author found that there is a direct correlation between pro-environmental attitudes and practical behaviour, although the intensity of this relationship varies depending on the cultural, economic and social context. For example, demographic factors such as age, education level and socioeconomic position significantly influence consumer choice. Furthermore, the adoption of sustainable behaviour is favoured by the presence of supportive policies and increased public awareness of environmental issues. In this sense, the sharing of identities, values and representations within a circumscribed context such as neighbourhoods seems a perspective to be valued and worthy of further investigation.

The most common theoretical model in which attitude-behaviour relations have been considered over the past three decades is the Theory of Planned Behaviour (TPB: Ajzen, 1991). Several studies have shown the theory’s value in predicting pro-environmental behaviour (Yuriev et al., 2019), with the crucial role played by behavioural intentions, considered as an indicator of an individual’s predisposition to try or how much effort a person is willing to make to perform a certain behaviour (Carrus et al., 2021; Hedlund-de Witt et al., 2014). However, intentions do not always predict behaviour. Many studies have in fact revealed a weak relationship between attitudes, intentions, and pro-environmental behaviour, which has led to the observation of an ‘attitude-behaviour gap’ (Wyss et al., 2022; Carrus et al., 2021).

Moreover, whilst a huge number of studies have explored and applied the TPB to specific sustainable behaviour, the same cannot be said in relation to sustainable lifestyles as a general concept. The concept of values, as approached by many environmental psychological studies, may help the understanding of this link.

Values are in fact often discussed in relation to environmental issues. Values change in desirability or importance at the individual level, making them a relevant source of variation between individuals, groups and communities. They are also motivational constructs that guide a person’s behaviour, but unlike specific goals and desires, they usually transcend situations. Values differ from attitudes in that attitudes are positive or negative evaluations of rather specific issues, whereas values tend to be more general indicators of people’s attitudes toward broader life circumstances, events or ideologies. Values, like attitudes, are generally moderately related to self-reported behaviour or behavioural intentions (Carrus et al., 2021).

With respect to the topic of sustainability and lifestyles, Gatersleben et al. (2010) explored the link between individual values and sustainable lifestyle indicators, highlighting how personal values influence daily choices and lifestyles, and how this has implications for environmental sustainability. Lifestyles are understood here as the set of everyday practises and choices; they reflect the interaction between values, personal habits and socio-cultural context. Individuals who adhere to pro-social and environmental values tend to integrate sustainable behaviour into their lifestyles, such as more moderate consumption, vegetarian or vegan food choices, and a focus on reuse and repair of goods. These authors note that there are barriers to switching to sustainable lifestyles, even for those with high pro-environmental values. These barriers may include high costs, lack of adequate infrastructure and cultural pressures toward consumerism. Therefore, concrete choices may do not depend solely on values, but also on external and structural factors (which are discussed in more detail in the next section). To foster sustainable lifestyles, it is then crucial to focus not only on individual values, but also on the social, cultural and economic contexts. Policies, education and awareness-raising campaigns can stimulate more sustainable values and behaviour, creating conditions that facilitate choices more compatible with the pursuit of environmental sustainability.

### 2.2 Collective believing

To develop our conceptual framework, we examined various theories and approaches in the context of the neighbourhood as a physical place, focusing on topics such as place attachment and place identity, as well as collective self-efficacy and environmental identity.

In general terms, identity processes are increasingly recognized as potential drivers of sustainable lifestyles and pro-environmental behaviours. This is true for different types of identity variables, such as social identity, environmental identity, and place identity. The impact of different types of identity factors on environmentally relevant behaviour has been extensively supported by empirical studies, theoretical models and meta-analyses (e.g., Carrus et al., 2005, 2014; Fornara et al., 2011; Fritsche et al., 2018; Vesely et al., 2021). Amongst these factors, place attachment plays an important role. Place attachment is defined as an emotional bond that people establish with specific places, based on personal experiences, social interactions and meanings attributed to those spaces. This concept is divided into two main dimensions: (a) emotional attachment, the sense of belonging and affection toward a place, (b) practical functionality, i.e., the value that the place provides in terms of resources and opportunities. This bond motivates residents to actively participate in the community and preserve local resources (Korpela, 2012), and is linked to the construction of individual and collective identities. The concept of place attachment has been in fact also considered as the affective component of a broader “Sense of Place” construct, together with place identity (which, in turn, represents a cognitive component; see Jorgensen and Stedman, 2001). It has been widely analysed in relation to the implementation of sustainable behaviour in small communities (both rural and urban), to which s can be assimilated. We argue that these concepts (place attachment, intention to adopt sustainable behaviour and identity process) are strongly interconnected and also work in synergy with lifestyles.

Indeed, Razem (2020) examined the link between place attachment and sustainable behaviour in neighbourhoods, emphasising how a strong emotional and psychological bond with the place of residence can stimulate actions in favour of sustainability. This author points out that place attachment can encourage residents to participate in actions aimed at improving the quality of place and preserving environmental and social well-being. For instance, people who are strongly attached to their neighbourhoods are more prone to take care of it, adopting sustainable behaviours such as recycling, waste reduction and resource conservation (as an act of responsibility to their place of living). Furthermore, a strong place attachment promotes community ties and cooperation amongst residents to address environmental and social issues (as a form of collective commitment). Finally, place attachment strengthens collective identity, spurring residents to protect and improve their environment, either through individual practises (such as urban gardening, for example) or community initiatives (such as local identity enhancement).

Place attachment encourages sustainable behaviour and lifestyles by promoting:

- Care for the local environment: Individuals who feel emotionally connected to a place are likely to engage in efforts to maintain its qualities by adopting practises such as recycling, conserving natural resources, and using sustainable transportation.
- Commitment to the community: A strong place attachment encourages residents to participate in community activities that enhance sustainability, such as urban gardening projects, cleanup initiatives, and the creation of shared spaces.
- Promotion of social cohesion: Positive relationships with neighbours and a sense of belonging strengthen the desire to contribute to the collective well-being and resilience of the neighbourhood.

Strengthening place attachment can be an effective strategy to promote sustainability in neighbourhoods. Designing urban environments that foster a sense of belonging, social relationships and access to natural and cultural resources can incentivise residents to engage in sustainable behaviour and actively participate in creating resilient communities.

Consequently, place attachment not only fosters people’s emotional well-being, but is also a powerful driver for the adoption of sustainable practises at the neighbourhood level. These are all aspects that favour the consolidation of sustainable and long-lasting lifestyles.

In this sense, attachment to place functions as a reinforcement for the sense of personal and collective identity and with that of mechanisms to foster collective efficacy (Fresque-Baxter and Armitage, 2012).

Collective self-efficacy is defined as a positive evaluation shared within a group (here a neighbourhood can be understood as a group, a collective with a shared identity) with respect to the group’s ability to organise and execute actions necessary to achieve certain goals. Group identification also triggers the mobilisation of personal resources to achieve group goals. However, this can be a double-edged sword in relation to the performance of pro-environmental behaviours (Brewer and Schneider, 1990). Whether the individual sees enacting a particular pro-environmental behaviour as bringing positive outcomes to his/her own group, then group identification will be a positive driver of sustainable lifestyle. On the contrary, when performing a specific action is seen as opposite to the interest of the group, then group identification will form a barrier to the adoption of sustainable lifestyles (Carrus et al., 2014). These processes have been nicely illustrated in both laboratory experiments on categorisation and cooperation in social dilemmas, as well in field studies on the social and cognitive processes at the basis of support for environmental policies (e.g., Carrus et al., 2005; Stoll-Kleemann, 2001).

These assumptions are also supported by the application of social identity theories to environmental issues (see Fritsche et al., 2018, for an integrative proposal). Social identity theory is based on the idea that individuals derive their identity from membership of particular social groups and thus tend to adhere to the norms of those groups (Terry et al., 1999). Environmental identity has been introduced in environmental psychology as a predictor of pro-environmental intentions. It is also emphasised that environmental identity can be influenced by previous pro-environmental actions (Sierra-Barón et al., 2023). Sparks and Shepherd (1992) demonstrated that one’s identity as a green consumer can predict pro-environmental intentions (namely intention to consume organic vegetables) over and above variables included in Ajzen’s (1991) TPB (see also Dean et al., 2012; Gatersleben et al., 2014; Lois et al., 2015; Lokhorst et al., 2014). What makes environmental identity a suitable focus of interventions designed to promote pro-environmental behaviour, including energy conservation and the adoption of new environmentally beneficial technologies, is the fact that identity appears to be malleable. In a similar vein, also the concept of a sense of community is defined as a feeling of belonging and attraction to a specific social group. The multidimensionality of the concept and its relevance to quality of life and community participation is relevant for our discussion, as the concept of sense of community typically includes spatial dimensions, identity issues, ideologies and general public representations and commonly shared fears. Its definition implies several levels of complexity. Amongst the aspects to be focused on, there is, for example, which dimension do people consider most to represent their reference community (the block, the neighbourhood, a symbolic place at the center of their social life), and the related sense of belonging that is determined by one’s community identity. In other cases, people may consider a “community” as a set of people with whom they share a religious orientation (e.g., the Buddhist community) or an ethnic or national group (the Italian community).

Although the concept of “sense of community” was originally introduced by Sarason in 1974, it was not until McMillan and Chavis (1986) seminal work that community psychologists began researching sense of community in a more systematic way. McMillan and Chavis (1986) defined the sense of community as “a feeling that members have of belonging, a feeling that members matter to one another and to the group, and a shared faith that members’ needs will be met through their commitment to be together” (p. 9). Despite the extensive attention devoted to the sense of community construct (Sarason, 1974; Pooley et al., 2005), Hill (1996) concluded that researchers have not succeeded in reaching an operational definition of psychological sense of community and that there is no universal agreement on the different dimensions that comprise this construct, thereby alluding to the notion that the construct is multidimensional.

In a 2008 paper, Manzini, Jégou and Penin linked sense of community to life studies as part of a project to promote sustainable lifestyles. The link between sense of community and sustainable lifestyles is a central theme to explain how people, through local and collective collaborations, can create more sustainable lifestyles. First and foremost, a sense of community is considered as a catalyst for change: a sense of community implies belonging, mutual support and a shared vision. These elements are key to promoting sustainable lifestyles, as people are more motivated to change their behaviour when they are part of a social network that supports such practises. Creative communities can foster empathy and respect for common resources, leading to a more conscious use of natural resources and reduction of waste.

As mentioned, community activities strengthen social bonds and promote the sharing of resources, decreasing the need for individualistic consumption and increasing collective well-being. A strong sense of community helps build common values that are fundamental to sustaining sustainability-oriented lifestyles. Values such as solidarity, social justice and environmental responsibility are more easily adopted and maintained in cohesive communities. A sense of community not only strengthens the social fabric, but is also a vehicle for implementing sustainable practises. Through cooperation and shared commitment, creative communities provide practical models for promoting sustainable lifestyles, reducing environmental pressure and improving collective well-being. Over time, these mutual interrelationship mechanisms can consolidate and support a long-term adoption of sustainable lifestyles.

All the constructs we briefly described are in some way interrelated and future research could lead to clarifying their function with respect to the development and consolidation of sustainability-oriented life studies. At the moment, such a complex theoretical framework has not always been accompanied by similar in-depth empirical investigation in specific areas, and we think that this conceptual work can be of help also in this direction.

### 2.3 Contextual factors

Beyond the individual and collective process we briefly discussed so far, a wide set of other contextual factors coming from disciplines like environmental science or and economics can be mentioned to the lifestyle change in real life contexts. These include aspects such as policy regulations, technology, infrastructure and markets and economic resources. These aspects have been in the focus of approaches that build on techno-economic, physical or environmental models in order ti to assess environmental and economic sustainability (e.g., Koljonen et al., 2012; Lehtilä and Koljonen, 2018; IEA, 2023; IPCC, 2022; EU, 2021). Studies with such an approach typically include a given set of scenario variants, occasionally with an effort to capture wider societal changes. Further examples of dealing with the relationships between contextual, collective and individual levels include efforts to numerically model lifestyles for their aggregated contributions to climate targets, through the use of computational models (e.g., van Sluisveld et al., 2016). However, deeper analysis building on individual or collective level factors is often lacking. The following examples of contextual factors for sustainable lifestyles and their connections to other orders of factors further highlight the need for an integrated approach that genuinely encompasses people in their living neighbourhoods.

Long-term strategies with targets can be described as the highest level of policies with an impact on lifestyle change, such as policy targets on climate neutrality set by the European Union for 2050 (EU, 2021; De Vita et al., 2021) and its Member States. There are relevant policies in many other sectors with potential impact on sustainable lifestyles as well, such as innovation policies, energy policies, agricultural policies, industrial policies, trade policies, educational policies etc.

Regulations or incentives set by public actors are one category of measures for implementing the types of policies described above (see, e.g., IEA, 2023). There are at least three mechanisms of regulations relevant to steer the sustainable choices in lifestyle changes: (i) directly, such as through building codes, renewable energy obligations etc. (ii) through economic incentives such as taxes or subsidies, aiming at making the sustainable choice attractive over the unsustainable, (iii) public hearings or information sharing mandated by regulation.

To discuss policies and regulations as contextual factors of lifestyle change^1^, the temporal dimension and the level of public bodies and authorities setting the regulations need to be underlined. More permanent impacts are usually achievable through long-lifetime investments incentivised, but also day-to-day behaviours can be impacted by proper regulation. Regulations can stem from European level bodies, as directives and acts, national legislation, as well as local decision-making processes (i.e., regional or municipal). Noteworthy, the decision-making parameters in control of municipal and national actors may very different between the countries and highly culture-specific, even within the European Union.

Technology typically contributes as an enabler and driver to the lifestyle changes, and the lifestyle changes are also closely related to technology available. Technological development contributes to the availability of alternatives to satisfy a service. On one hand, technology development is driven by market demand and its dynamics; on the other hand, the development can be boosted by R&D&I policy measures by public bodies (e.g., IPCC, 2022). During the last decades, considerable steps in many areas relevant for lifestyle changes have been taken at least in development and implementation of Electric Vehicles, renewable energy generation such as and wind and solar power, and information and communications technologies.

For the framework proposed in this paper, the size of a technological investment is also of importance. For example, a national-scale centralised power or heat production plant would mostly affect the municipalities or neighbourhoods indirectly. On the other end of the spectrum, citizens or collectives in modern societies can make decisions quite independently on whether or not to purchase an electric vehicle or an e-bike, or whether to implement an energy renovation to their houses or other facilities in their ownership and/or control. Naturally, economic restrictions make some novel solutions unaffordable for many: this is one example of the interplay between the groups of contextual factors. However, technological development over time also pushes the costs down and enhances the spectrum of alternatives (e.g., IEA, 2023). For example, analysis of a sustainable lifestyle, e.g., in emission-free transportation 20 or even 10 years ago would be essentially different than with the technology portfolio available today.

Infrastructure represents another category of contextual factors closely related to technology. Here, we especially discuss technical infrastructure (and not social etc.). Infrastructural factors appear to the citizens, for example, as power transmission lines, district heating pipelines, and vehicle charging/fuelling networks. As a concrete example, the planning and implementation of cycling and walking roads for emission-free transport, more walkable streets, green sidewalks, pedestrian zones, green areas and similar infrastructures are factors in hands of municipal planners that may be crucial to enable lifestyle changes, even beyond the single specific behaviour that they afford (such as, cycling or waling).

Also more generally, one can argue that the infrastructural factors are, in essence, a playfield of society rather than of individuals. However, the position of individuals and neighbourhoods could and should be reflected in infrastructures through bottom-up and participated policy processes.

With infrastructures, lifetimes are typically long. For example, urban form is very permanent for old cities. However, metropolitan regions as well as other “larger cities” are growing all the time due to urbanisation dynamics. Therefore, there can be very stable regions and also totally new suburb regions inside the city. Hence, more sustainable lifestyles are achievable also through the planning and changing of infrastructures. On the other hand, reforms in infrastructures in old cities can have practical difficulties, being often very costly and time-consuming if not even impossible. As a contextual factor, economic resources are often discussed under framework of market-based processes, as a mechanism to implement exchange of resources in economy.

These aspects seem close to the proposal of the French geographer Moine (2006) who described the territory as a complex system, consisting not only of a physical space, but including social, economic, cultural and environmental relations. This approach views territory not merely as a physical space but as a dynamic set of relationships and flows. His approach emphasises the interconnection between the elements that make up the territory and the dynamics that transform it over time.

This author emphasises the importance of abandoning reductionist views in order to adopt a systemic perspective that allows for a better understanding of territorial phenomena. A complex system is characterised by (a) interdependence between elements, (b) non-linearity in relationships, which makes it difficult to predict outcomes, (c) emergence, i.e., the appearance of unpredictable phenomena resulting from the interaction of the elements, (d) adaptability, which allows the system to evolve in response to external changes.

In this framework, the territory is seen as a dynamic entity that is constructed through the continuous interaction between actors (individuals, groups, institutions) and their environment. Although Moine did not deal specifically with the lifestyle concept, his model is useful in providing a complex and modern view of this topic and it seems completely relevant for an application to neighbourhoods’ life.

Looking for a more classical reference in the social sciences, from a macro perspective (e.g., Bronfenbrenner, 1979), economic resources are related to the overall financial viability of a community or a nation. On one hand, economic resources are beneficial or even necessary to enable investments for sustainable lifestyles. They can be measured, e.g., by economic development indexes of GDP and be linked to factors such as productivity, employment, demographic development and export–import balance of the country or region (e.g., IPCC, 2022). On the other hand, the level of consumption and use of natural resources, including overuses conflicting with sustainable limits, are typically closely linked to the growth of GDP.

From a more micro-level perspective, energy poverty is also an important concept related, essentially to the lack of economic resources to cover increasing energy expenses in the energy transition phase and in a time of energy supply crisis like the ones Europe is currently facing. Related to this, a new phenomena is also “transport poverty,” which is especially seen in the rural areas with no or limited access for public transport. To understand the dynamics of these emerging phenomena, a evidence-based approaches pointing to indications of high social and economic status enabling high-impact lifestyles are also worth mentioning here (e.g., Schwarzinger et al., 2019).

## 3 Conclusions: basic assumptions for neighbourhood interventions and avenues for future research

The concept of “lifestyle” plays an important role in social science and humanities research on energy and sustainable behaviour. The publications and models available to date suffer from excessive disciplinary fragmentation. In this paper, we have attempted to integrate a new, all-encompassing perspective on lifestyles as applicable to this context into a single model. Despite the methodological heterogeneity and pluralism of research traditions that make the search for common theory and research lines complex, this attempt may bring novelty and dynamism to the field. In fact, a better and deeper discussion and understanding of terminologies, definitions and methods seems necessary for a constructive discourse. We do not explicitly advocate any standardisation of lifestyle-related research methods or the reduction of future empirical studies to a few possible variants of lifestyle research. However, we think that other scholars using the lifestyle concept may want to focus their work to the three dimensions identified in this review. This would provide greater transparency on the specific lifestyle concept to which a particular research or publication applies, and help other researchers to quickly identify relevant work and compare methods and results. Presumably, the “lifestyles” perspective will also gain importance in public decision-making, with the aim of establishing new policies on pro-environmental behaviour and regulations. Our three dimensions indicate future avenues in which lifestyles research could contribute to the grand challenge of mitigating climate change and adapting human lives to it. The neighbourhood, as a fundamental unit of living habitat in many current human societies, can play a pivotal role as the most proximal and meaningful social, economic, environmental and institutional setting for citizens, in which sustainable lifestyles can be effectively promoted. Within this microsystem, the development of a ‘sustainability culture’ encompassing both material conditions and psychological factors may encourage the adoption of pro-environmental lifestyles and behaviours at both an individual and collective level. According to the ecological theory of human development (Bronfenbrenner, 1979), the crucial aspect for the development of the individual is the person’s experience and perception of the social and physical context with which it interacts, which points out the relevance of a number of individual-level psychological factors and social group variables in shaping the adoption of sustainable behaviours in one’s own daily life context. However, since lifestyle models do not take shape in a social vacuum, we shall assume that contextual factors can also interact with further collective factors involving the meso-level system of societal structures. Indeed, they are formed through social interactions within and between groups, also as a consequence of social learning processes that, in turn, might empower individuals to change their institutional, organisational, political and physical milieu. To fully understand sustainable lifestyles, a multidisciplinary approach is “mandatory.” Investigations on this topic are necessarily based on conceptions deriving from a broad spectrum of social science and disciplines including economics, sociology, political science and psychology (Manzini et al., 2008). The sustainable lifestyle represents indeed a common study area where the actualization of individuals’ needs is confronted with the limits of systemic interference with the Earth’s complex systems. These limits might be faced in the form of national or local legislation due to implementation of climate and energy policies, or due to changes taking place locally. They may include for example, structural economic changes, changes within the local infrastructure or changes felt in the natural environment due to climate change. Also, it is important to underline not only the potential tension between individual needs and planetary limits, but also those limits and constraints that the society at the administrative, normative or institutional level might be setting to individuals, groups and communities. Thus, a worthy issue to address in this field of investigation is how the interplay between individual and collective factors take shape in defining sustainable lifestyles, positive engagement and transformative action within specific neighbourhoods. In order to understand how broadening the perspective of sustainable development in daily life contexts at a systemic micro-level may trigger action in local communities, and how this kind of learning may encourage political bottom-up driven decisions, we need to understand and use local knowledge, challenge accepted norms, explore the cultural background of energy practises and stimulate the co-creation of cultures/narratives of change in the variety of case human life contexts that can shape the individual experience, even beyond their strict personal and psychological characteristics. This might also imply the need of broadening the perspective from single domains at individual level factors, as well as broadening the perspective from the choices and wellbeing of single individuals toward those of the communities or the society as a whole, also in line with more social and collective approaches to human social interaction such as, for example the social representation theory propose by the Moscovici (1961). This learning process at micro level allows a better understanding of systems dynamics of a society at this level, in terms of climate change awareness and decision making in the transforming process. To the purposes of this paper, future specific empirical actions could therefore consist in a multi-method assessments of the individual, social and cultural factors underpinning the adoption of a more sustainable lifestyle in given local situations, across different domains, across the different spatial context of the daily life, and to what extent these patterns could emerge as stable trends through the time as a result of transformative learning processes. This would also imply to include increased awareness and understanding of climate change dynamics and connections at different levels. Such increased awareness may not directly change people’s lifestyles in the short term but rather promote changing intentions that might trigger action at an individual or neighbourhood levels, in the long term. In sum, in the present paper we propose an approach that defines sustainable lifestyle as that which actually reduces environmental impacts or at least that has a purpose and subjective meaning in that direction, and which might exert its effects in the longer term and through aggregated efforts of individuals, groups and communities.

## Footnotes

^1^There is no reference to a specific set of behaviours here; the discourse is general and relates to a macro-level, in line with the eco-systemic approach.
